# Pyomyositis in an Immunocompetent Adult Man

**DOI:** 10.7759/cureus.104844

**Published:** 2026-03-07

**Authors:** Mohammad Ayoub, Humam Rajha, Moath A Albdyrat, Salah Almughalles, Abdulqadir J Nashwan

**Affiliations:** 1 Department of Internal Medicine, Hamad Medical Corporation, Doha, QAT; 2 College of Medicine, QU Health, Qatar University, Doha, QAT; 3 Department of Radiology, Hamad Medical Corporation, Doha, QAT; 4 Department of Nursing and Midwifery Research, Hamad Medical Corporation, Doha, QAT

**Keywords:** computed tomography, iliopsoas abscess, image-guided drainage, intramuscular infection, pyomyositis

## Abstract

Pyomyositis is an uncommon bacterial infection of skeletal muscle, most frequently affecting large lower-limb muscle groups and often associated with abscess formation. While historically prevalent in tropical regions, cases are increasingly reported in temperate climates. Diagnosis is often delayed due to nonspecific early symptoms and deep muscle involvement. We report a 24-year-old previously healthy male presenting with a one-month history of intermittent dull right lower back pain, antecedent fever with chills, intermittent low-grade fever, night sweats, and 10 kg weight loss over two months. Examination revealed a firm, tender swelling in the right flank. Laboratory studies showed anemia (hemoglobin 10.6 g/dL), markedly elevated CRP (181.9 mg/L) and erythrocyte sedimentation rate (47 mm/hr), and a normal leukocyte count. Contrast-enhanced CT demonstrated a bulky right iliopsoas muscle with multiple abscesses. Initial management included IV ceftriaxone, anticoagulation prophylaxis, and supportive care, with planned image-guided drainage. Follow-up imaging and inflammatory markers at two weeks showed marked abscess regression, prompting step-down to oral therapy. The patient later re-presented with recurrent flank swelling; CT revealed a new posterior abdominal wall intramuscular abscess. Ultrasound-guided aspiration yielded sterile pus, negative for acid-fast bacilli. He completed a 21-day course of oral amoxicillin/clavulanate with clinical improvement. This case highlights the diagnostic challenge of pyomyositis in immunocompetent individuals, in whom constitutional symptoms and deep muscle involvement can obscure early recognition. Cross-sectional imaging is critical for diagnosis, particularly when clinical signs are subtle. Optimal management requires a multidisciplinary approach integrating appropriately dosed antibiotics of adequate duration, timely image-guided drainage, and vigilant follow-up to prevent recurrence. Early recognition and intervention are essential to reduce morbidity in this potentially serious infection.

## Introduction

Pyomyositis is a rare condition defined as an acute or subacute bacterial infection of skeletal muscle, often characterized by abscess formation within large striated muscle groups, particularly those of the lower limbs [1]. Although historically associated with tropical regions, more cases are now being reported in temperate climates. This trend likely reflects heightened clinical awareness, an increasing number of immunocompromised patients, and improved diagnostic techniques [2]. While global prevalence statistics remain variable, recent literature suggests a rising incidence in temperate zones, with mortality rates ranging from 1% to 10% depending on the promptness of diagnosis and the presence of systemic sepsis [3,4]. In culture-positive cases, *Staphylococcus aureus* remains the predominant pathogen, accounting for approximately 70% of infections in tropical settings, while Group A streptococcus accounts for 1-5% [5,6].

The pathogenesis of pyomyositis is thought to involve hematogenous spread of bacteria, often secondary to bacteremia, dental procedures, or urinary tract infections. In immunocompromised patients, pyomyositis is frequently opportunistic and linked to broader systemic vulnerabilities. In immunocompetent hosts, pathogenesis typically involves hematogenous seeding of a muscle bed rendered susceptible by unrecognized trauma or strenuous exertion. Risk factors include immunodeficiency (e.g., HIV, diabetes mellitus, and hematologic malignancies), trauma, and strenuous exercise [7]. Despite these associations, pyomyositis can also occur in immunocompetent individuals, posing diagnostic challenges due to its nonspecific early symptoms, such as localized muscle pain, fever, and swelling, which often mimic other musculoskeletal or septic conditions.

Clinically, the condition progresses through three stages: an initial invasive stage marked by localized cramping and induration; a suppurative stage characterized by frank abscess formation, fever, and significant localized pain; and a final systemic stage marked by bacteremia and multiorgan failure. Delayed diagnosis is common, with studies reporting a median time of seven to 10 days to identification after admission. This delay is partly due to the deep-seated location of affected muscles and the absence of overt local signs [8], presenting significant diagnostic pitfalls. The retroperitoneal location of muscles such as the psoas or iliacus means the overlying fascia masks superficial erythema or fluctuance, frequently leading clinicians to misdiagnose the condition as a muscle strain, spinal pathology, or intra-abdominal emergency.

Treatment hinges on prompt abscess drainage and targeted antibiotic therapy, typically requiring prolonged IV administration to prevent progression to systemic sepsis. Given its potential for rapid deterioration, early recognition is critical to reducing morbidity.

## Case presentation

A 24-year-old male presented with a one-month history of intermittent, dull, aching pain in the right lower back. He is a daily smoker with no significant past medical history. He reported an episode of fever with chills one month prior to presentation, followed by intermittent low-grade fever, night sweats, and a weight loss of 10 kg over two months. He denied cough, sputum production, abdominal pain, dysuria, pyuria, altered bowel habits, blood or mucus in stools, melena, or prior surgeries.

On initial examination, the patient was afebrile, with a tympanic temperature of 37.5°C, a heart rate of 94 bpm, a respiratory rate of 16 breaths per minute, a blood pressure of 130/65 mmHg, and an oxygen saturation of 99% on room air. His weight was 60 kg. General examination revealed no pallor, icterus, cyanosis, clubbing, edema, or lymphadenopathy. Respiratory and cardiovascular examinations were unremarkable, with equal bilateral breath sounds and normal heart sounds without murmurs. Abdominal examination revealed a soft, nontender abdomen with no organomegaly or shifting dullness; however, a firm, tender swelling was palpable over the right flank. Dermatological evaluation was unremarkable, with no occult lesions or skin changes. Neurological examination revealed no signs of meningeal irritation or focal deficits.

These findings suggested a localized process in the right flank, potentially inflammatory or infectious. Differential diagnoses included renal, perinephric, or psoas abscess, vertebral osteomyelitis, or extrapulmonary tuberculosis.

**Figure 1:**
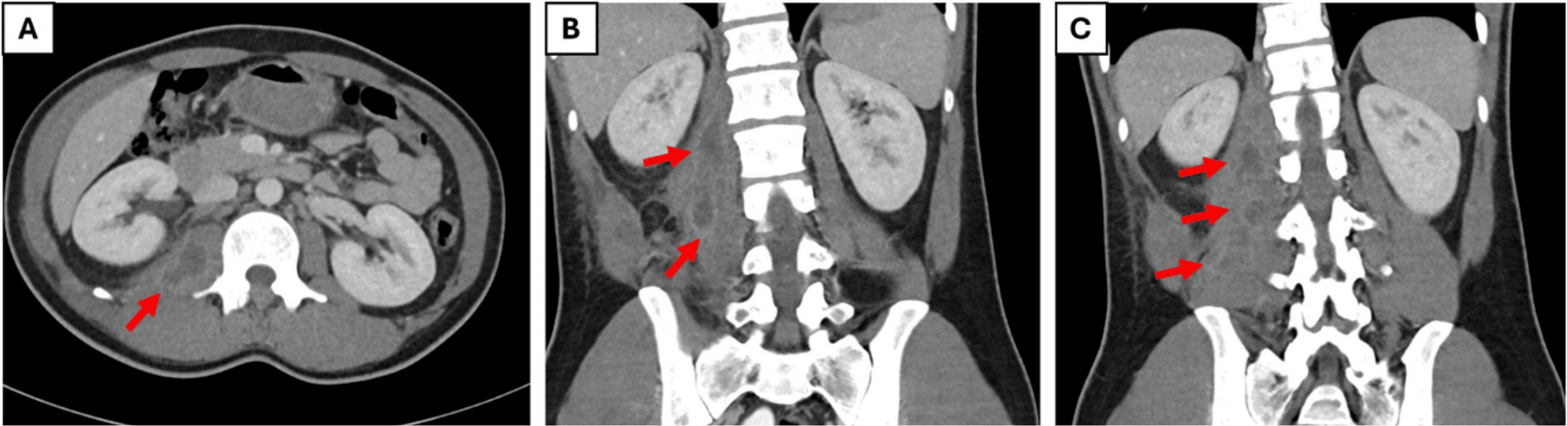
Selected axial (A) and coronal (B, C) contrast-enhanced CT images showing a multiloculated abscess tracking along the entire swollen right psoas muscle into the iliacus, with enhancing septations and surrounding inflammatory changes

Diagnostic workup included a normal chest X-ray and contrast-enhanced CT of the abdomen, which revealed a bulky right iliopsoas muscle with heterogeneous enhancement and multiple abscesses, the largest measuring 55 × 27 mm (Figure 1).

The underlying bone and intervertebral disc spaces were intact. Mild right ureteral dilatation and a small right-sided pleural effusion were noted, likely secondary to adjacent inflammation. Laboratory results showed anemia (hemoglobin 10.6 g/dL), elevated inflammatory markers (CRP 181.9 mg/L, erythrocyte sedimentation rate (ESR) 47 mm/hr), and a normal white blood cell count (10.6 × 10³/µL). Liver and renal function tests were within normal limits. Additional investigations are summarized in Table 1.

**Table 1:** Laboratory findings on admission and follow-up The marked elevation in CRP (181.9 mg/L), despite a normal WBC count (10.6 × 10³/µL), underscored the severity of the deep-seated inflammatory process and served as the primary biochemical marker for monitoring treatment response. ALP, alkaline phosphatase; ALT, alanine aminotransferase; ANC, absolute neutrophil count; APTT, activated partial thromboplastin time; AST, aspartate aminotransferase; Hct, hematocrit; Hgb, hemoglobin; INR, international normalized ratio

Investigation	Admission value	Follow-up value	Reference range
General hematology			
WBC	10.6	9.1	4.0-10.0 × 10⁹/L
RBC	4.3	4.5	4.5-5.5 × 10¹²/L
Hgb	11.3	12.5	13.0-18.0 g/dL
Hct	36.1	38.3	38-50%
Platelet	431	327	150-400 × 10⁹/L
ANC	7.4	4	2.0-7.5 × 10⁹/L
Coagulation			
Prothrombin time	15.7	13.1	11.0-13.5 seconds
INR	1.4	1.1	0.8-1.2
APTT	37.7	40.5	25-35 seconds
Blood chemistry			
Urea	3.9	3.9	2.5-6.0 mmol/L
Creatinine	66	55	60-110 µmol/L
Sodium	139	139	135-145 mmol/L
Potassium	4.1	4.2	3.5-5.0 mmol/L
Chloride	99	105	95-105 mmol/L
Bicarbonate	27	24	22-28 mmol/L
Calcium	2.38	-	2.20-2.60 mmol/L
Bilirubin, total	8	4	5-20 µmol/L
Total protein	88	71	60-80 g/L
Albumin	35	34	35-55 g/L
ALP	100	70	40-150 U/L
ALT	6	10	7-56 U/L
AST	13	13	10-40 U/L
Glucose	6.7	-	3.9-5.5 mmol/L
CRP	181.9	37.3	0-10 mg/L

**Figure 2:**
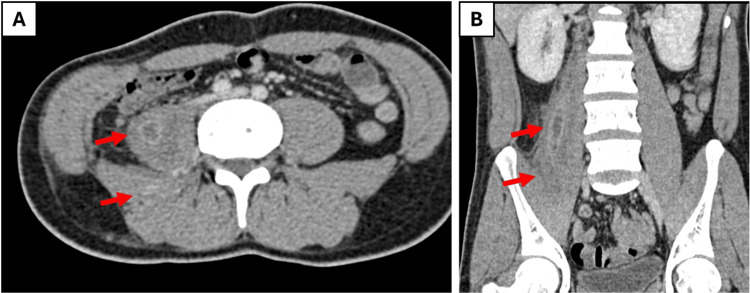
Follow-up (two-week interval) selected axial (A) and coronal (B) contrast-enhanced CT images demonstrate significant regression of the previously noted multiloculated abscess within the right psoas and iliacus muscles The collection has markedly decreased in size, with resolution of muscle swelling and surrounding inflammatory changes.

The patient was admitted and started on IV ceftriaxone (2 g daily) and subcutaneous enoxaparin (40 mg daily) for venous thromboembolism prophylaxis, along with supportive care, including sodium chloride nebulization and as-needed paracetamol. Further investigations, including blood cultures, sputum acid-fast bacilli (AFB), and HbA1c, were ordered to clarify the etiology. A CT-guided aspiration was initially planned but deferred after follow-up imaging at two weeks showed a significant reduction in abscess size and inflammatory markers (Figure 2), prompting discharge on oral antibiotics (amoxicillin/clavulanate 1 g twice daily and metronidazole 400 mg three times daily) with scheduled outpatient follow-up. However, the patient returned within weeks, reporting increased right flank swelling and pain, necessitating readmission.

**Figure 3:**
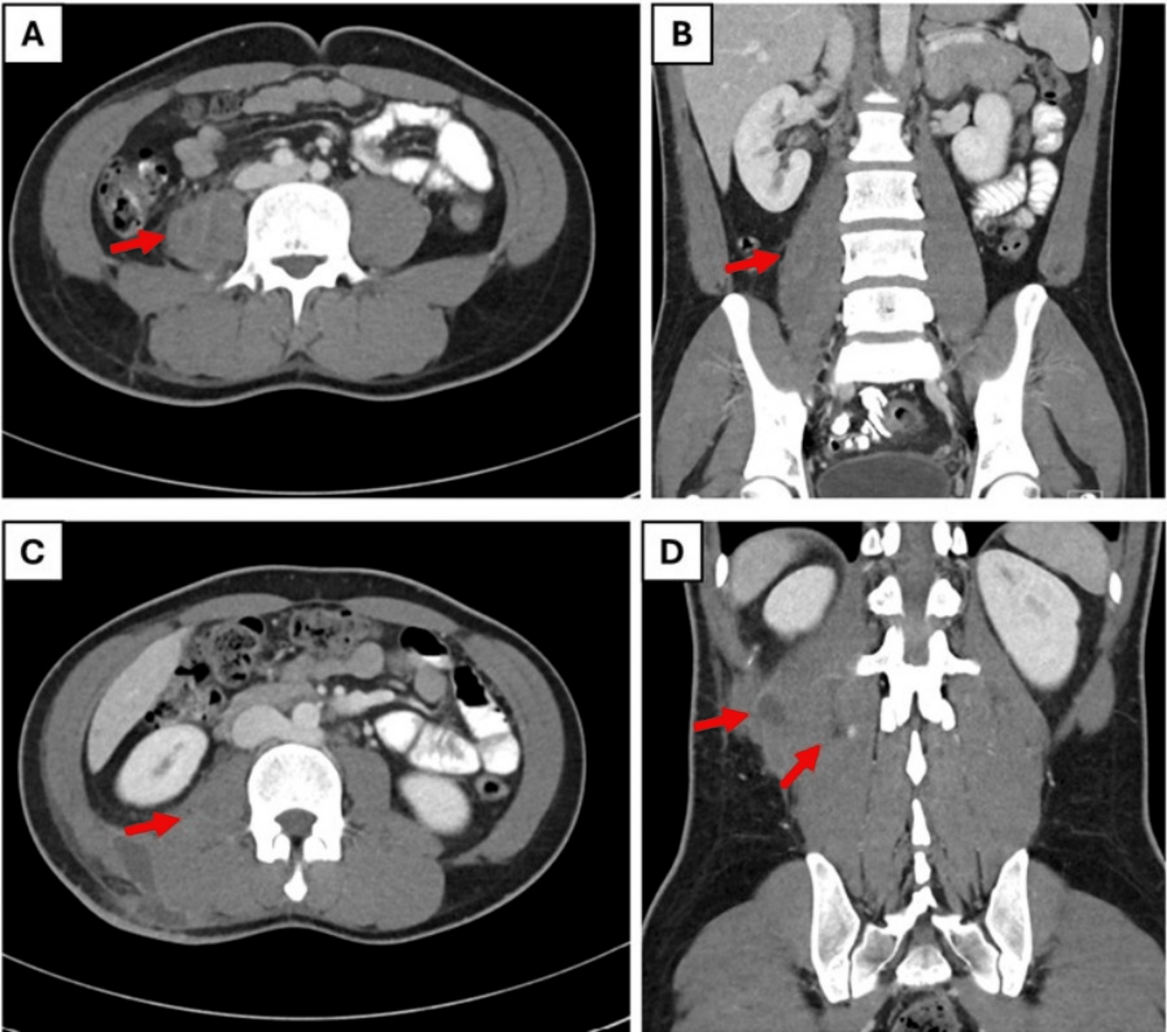
Third follow-up (one-month interval) selected axial (A, C) and coronal (B, D) contrast-enhanced CT images reveal continued regression of the previous right psoas and iliacus abscess, with resolution of associated muscle swelling A new multiloculated fluid collection is now identified along the right paraspinal muscle plane, suggesting possible extension or recurrence in a different myofascial compartment.

During his subsequent admission one month later, physical examination revealed a firm, tender swelling with erythema in the right lower back. Repeat CT imaging confirmed a right posterior abdominal wall intramuscular abscess (measuring 100 × 12 × 12 mm) with significant regression of the prior psoas abscess, which had decreased from its initial 55 × 27 mm (Figure 3). Laboratory findings showed improvement in inflammatory markers (CRP 37.3 mg/L, ESR 26 mm/hr) and hemoglobin (12.5 g/dL) (Table 1). Interventional radiology (IR) was consulted. Although the patient initially declined hospitalization for a planned ultrasound-guided aspiration, he returned a week later and consented to the procedure. IR performed an ultrasound-guided aspiration of the right lumbar subcutaneous collection, yielding AFB-negative fluid and negative cultures. Post-procedure, the patient was stable, and the swelling reduced significantly.

The patient was discharged the next day in stable condition, with a prescription for amoxicillin/clavulanate (1 g twice daily) to complete a 21-day course and as-needed paracetamol. Follow-up with internal medicine was arranged in two weeks to assess for recurrence.

## Discussion

The clinical presentation in this case, as subacute, dull, aching lower back pain evolving over one month, accompanied by antecedent fever with chills, intermittent low-grade fever, night sweats, and significant weight loss (10 kg), exemplifies the insidious onset of pyomyositis. Pyomyositis typically progresses through three stages: (1) invasive (localized pain/swelling without systemic signs); (2) suppurative (abscess formation with fever/malaise); and (3) disseminated (sepsis/multiorgan dysfunction). This case aligns with the suppurative phase, though the subacute timeline and constitutional symptoms (e.g., weight loss and night sweats) underscore the variability in progression, particularly in immunocompetent hosts [9]. While pyomyositis in immunocompromised patients is frequently opportunistic and linked to broader systemic vulnerabilities, pathogenesis in immunocompetent hosts typically involves hematogenous spread of bacteria that seeds a muscle bed rendered susceptible by unrecognized trauma or strenuous exercise.

Such nonspecific systemic symptoms, coupled with the deep anatomical location of the psoas muscle, frequently lead to diagnostic delays, with studies reporting a median time to identification of seven to 10 days after initial presentation [8]. The absence of overt localizing signs, such as erythema or fluctuance, in the early stages, and the deep anatomical location of the psoas muscle, frequently lead clinicians to initially misdiagnose the condition as a musculoskeletal strain or spinal pathology. Ultimately, the discovery of a firm, tender swelling in the right flank during physical examination proved vital, shifting the diagnostic focus toward an inflammatory or infectious process and underscoring the critical importance of meticulous serial examination when systemic symptoms accompany persistent localized pain.

Diagnostic imaging, such as contrast-enhanced CT, was instrumental in confirming the diagnosis, revealing a bulky right iliopsoas muscle with heterogeneous enhancement and multiple abscesses (Figure 1). This finding is characteristic of pyomyositis [10]. Laboratory investigations supported a significant inflammatory process, evidenced by a marked elevation in CRP (172.3 mg/L) and ESR (47 mm/hr), along with anemia (hemoglobin, 10.8 g/dL). Notably, the white blood cell count remained within normal limits (10.0 × 10³/uL), highlighting the limitation of relying solely on leukocytosis as a marker for deep-seated musculoskeletal infection [8]. In this case, the markedly elevated CRP and ESR provided a more accurate reflection of the systemic inflammatory burden.

This diagnostic weight enabled us to maintain a high suspicion of an infectious process despite the absence of a high white cell count, a common pitfall that can lead to diagnostic delays in pyomyositis. The diagnostic workup rightly included efforts to differentiate pyogenic from tuberculous etiologies via sputum AFB testing and cultures, given the patient’s constitutional symptoms and the epidemiological consideration of tuberculosis, particularly in regions of endemicity. However, the practical challenge of obtaining timely microbiological confirmation was evident: initial CT-guided aspiration was deferred, and culture results remained pending at key decision points. However, the low yield of blood cultures (5-30% positivity) underscores the necessity of abscess aspiration with Gram stain, aerobic/anaerobic cultures, and fungal/TB testing (e.g., GeneXpert and MGIT) for definitive diagnosis, as up to 30% of pus cultures may be negative [9].

Management commenced appropriately with empiric IV antibiotic therapy targeting common pyogenic pathogens; ceftriaxone provides reliable coverage against *S. aureus *and streptococci. The choice of ceftriaxone was justified by the severity of the patient’s systemic symptoms and local microbial patterns, which favor third-generation cephalosporins as a robust empiric starting point for community-acquired pyogenic infections in our region. The subsequent transition to oral amoxicillin/clavulanate, while a logical step-down option, preceded definitive source control. The recurrence of symptoms and swelling shortly after discharge strongly suggests either inadequate drainage of the abscess or an insufficient duration of antimicrobial therapy at that stage. Current guidelines recommend prolonged IV antibiotic courses, typically three to four weeks, for pyomyositis to prevent complications and recurrence, where mortality rates in late-stage pyomyositis remain high (up to 30%) [11,12].

The initial regression of the psoas abscess on follow-up imaging (Figure 2), while encouraging, was followed by the emergence of a new posterior abdominal wall intramuscular collection (Figure 3), demonstrating the dynamic nature of these infections and the necessity for vigilant monitoring. The eventual ultrasound-guided aspiration provided symptomatic relief and yielded AFB-negative fluid, leaning toward a pyogenic etiology, though definitive culture results remained crucial for targeted therapy. This sequence underscores the essential role of a multidisciplinary approach that integrates IR for timely drainage and infectious disease expertise for antimicrobial stewardship, particularly when cultures are delayed or negative. Negative cultures significantly complicate the clinical decision-making process by hindering the transition to narrow-spectrum, targeted therapy, thereby prolonging reliance on empiric broad-spectrum regimens. In the absence of culture sensitivities, patient management must rely heavily on a multidisciplinary approach and the meticulous serial monitoring of inflammatory markers to gauge treatment efficacy, as was done in this case.

Etiological considerations centered predominantly on pyogenic bacteria, given the AFB-negative aspirate and the absence of classic risk factors for tuberculosis. The 10 kg weight loss and night sweats are indeed classic red flags for tuberculosis, and a single negative AFB stain from the aspirate is insufficient to definitively rule out TB. Furthermore, it must be explicitly acknowledged that the sterile culture results were highly likely influenced by the initiation of broad-spectrum antibiotics. The patient had received a course of IV ceftriaxone followed by oral amoxicillin/clavulanate well before the definitive ultrasound-guided aspiration, making the sterile yield likely a consequence of this extensive prior antimicrobial exposure rather than an indicator of true sterility. *S. aureus *remains the most probable causative agent, although Group A streptococcus or other organisms cannot be excluded without culture confirmation [13]. Given this limitation, attributing the infection to *S. aureus *remains a presumptive, although highly probable, clinical deduction rather than one based on direct microbiological evidence.

While tuberculous psoas abscesses are strongly associated with underlying vertebral osteomyelitis (Pott’s disease) and immunocompromise, rare cases do occur in immunocompetent hosts, making it a necessary, albeit less likely, differential in this context [14]. Our clinical decision to lean toward a pyogenic etiology was largely driven by the intact vertebral bone and disc spaces on CT (ruling out Pott’s disease, which is typically associated with tuberculous psoas abscesses), the normal chest X-ray, and the patient’s rapid initial response to broad-spectrum pyogenic antibiotics. Other potential sources, such as vertebral osteomyelitis or renal abscess, were reasonably excluded by the intact bone and disc spaces on CT and unremarkable urinalysis, respectively. However, TB remained a necessary differential requiring close longitudinal monitoring.

The patient’s course emphasizes critical aspects of follow-up and prognosis in pyomyositis. Early recurrence following initial discharge highlights the vulnerability to relapse if source control is incomplete or antibiotic therapy is truncated prematurely. Close outpatient monitoring, including serial assessment of inflammatory markers (CRP and ESR) and clinical examination, is paramount. Repeat imaging may be warranted if symptoms persist or recur to assess abscess resolution or identify new collections, ensuring complete resolution and preventing long-term sequelae. Furthermore, studies on long-term outcomes in pyomyositis are highly encouraged to improve our understanding of the disease trajectory and to refine longitudinal management strategies. While surgical debridement is generally reserved for extensive or refractory cases that are not amenable to percutaneous drainage, the significant symptomatic improvement after aspiration in this case supports the effectiveness of minimally invasive approaches when feasible and timely [15]. Prolonged oral antibiotic therapy (21 days total in this instance) aims to eradicate residual infection and prevent complications such as bacteremia, sepsis, or fistula formation.

## Conclusions

This case serves as a salient reminder of the diagnostic intricacies of pyomyositis, particularly when it presents as a psoas abscess. Clinicians must maintain a high index of suspicion for this entity in patients presenting with subacute back or flank pain accompanied by systemic symptoms like fever and weight loss, even in the absence of classic risk factors or overt immunosuppression. Early utilization of advanced cross-sectional imaging (CT or MRI) is indispensable for timely diagnosis, as physical findings can be subtle or delayed. Effective management hinges on a multidisciplinary strategy combining prolonged, pathogen-directed antimicrobial therapy initiated empirically based on local epidemiology and patient factors, with prompt radiological or surgical drainage of accessible abscesses. Vigilant follow-up is essential for promptly detecting and managing recurrence or complications. This comprehensive approach, emphasizing early recognition, the necessity of definitive source control to prevent recurrence (as demonstrated by the initial relapse in this case), adequate antibiotic duration, and collaborative care, remains fundamental to optimizing outcomes and minimizing morbidity in this potentially serious infection.
